# Study on knowledge, practices, and attitudes toward decentralized clinical trials among the clinical trial practitioners in China

**DOI:** 10.3389/fmed.2025.1513975

**Published:** 2025-04-17

**Authors:** Yuanyuan Shi, Yujian Bao, Yu Pu, Junhao Jiang, Bo Yan, Hang Zheng

**Affiliations:** ^1^School of Pharmacy, Chongqing Medical University, Chongqing, China; ^2^Medical Imaging Key Laboratory of Sichuan Province, Affiliated Hospital of North Sichuan Medical College, Nanchong, China

**Keywords:** decentralized clinical trials, DCT, remote clinical trial, survey, questionnaire

## Abstract

**Background:**

Studies on the application of decentralized clinical trials (DCTs) in China are limited. This study aimed to investigate the knowledge, practices, and attitudes of clinical trial practitioners in China toward DCTs.

**Method:**

An anonymous cross-sectional study was conducted from November 1st to November 30th, 2023. A total of 621 valid questionnaires were collected, including 227 completed by Clinical Research Associates (CRAs), 150 by Clinical Research Coordinators (CRCs), and 244 by Program Managers (PMs).

**Results:**

The majority of respondents possessed basic knowledge of DCTs and showed a high willingness to participate, but their practice experience was limited, with a relatively high level of practice experience in internet recruitment. Respondents were more interested in the improvement of patients’ rights brought by DCTs, such as more opportunities for clinical trials. Technical barriers and data reliability were the main barriers to implementation.

**Conclusion:**

Clinical trial practitioners need to enhance their technical skills and knowledge about DCTs, enhance the overall level of the industry, and promote the better implementation and application of DCTs in China.

## 1 Introduction

Clinical trials consume a significant amount of time and resources in drug development. In the past, these trials were site-centered, requiring participants to frequently visit the trial site for treatment evaluation and data collection, which imposed a significant burden and hindered recruitment and retention efforts (1). In recent years, decentralized clinical trials (DCTs) have grown in popularity as clinical trials have shifted to more efficient, flexible, and patient-centered approaches (2). DCTs involve the use of digital technologies and remote monitoring methods to reduce or eliminate reliance on traditional centralized trial sites such as hospitals or research centers (3). This approach minimizes disruptions to participants’ daily lives, enables recruitment of a more diverse group of participants, enhances participation rates and data authenticity, and reduces costs (4).

The COVID-19 pandemic forced regulators to issue provisional guidances (5–7) to ensure the normal conduct of clinical trials by using some decentralized elements (8, 9). Subsequently, the US Food and Drug Administration (FDA), the European Medicines Agency (EMA), and regulatory agencies from other countries have all developed formal guidances for DCTs (10–12). At the same time, the quantity of DCTs are also rapidly developing. Based on the ClinicalTrials.gov database, Sato found that the number of trials incorporating DCT components has steadily increased since the first study in 2001, and this growth trend has been more significant since 2020 (13).

China National Medical Products Administration (NMPA) has been actively promoting the development of DCTs. In 2021, the Center for Drug Evaluation (CDE) of the NMPA issued the “Guidance on Clinical Value-Oriented Clinical Development of Anticancer Drugs,” which encouraged the integration of DCTs elements into trials for anticancer drugs (14). Subsequently, DCT elements were incorporated into the “Guidance for the Use of Real-World Evidence to Support Drug Development” (15) and “Technical Guidance for the Implementation of Patient-Centered Drug Clinical Trials (Trial)” (16). In October 2023, the Beijing NMPA took the lead in releasing the Implementation Plan for DCTs Pilot Work, initiating DCTs pilot projects nationwide. Building on this, in May 2024, the CDE released the “Technical Guidance for the Application of Decentralized Clinical Trials in the Clinical Development of Drugs for Rare Diseases,” marking Chinese first technical guidance related to DCTs (17). It was evident that the development of DCTs not only meets the practical needs of enterprises but also aligns with the focus and guidance of policy initiatives. The future application scenarios for DCTs are set to expand further.

As the front-line practice participants of clinical trials, clinical trial practitioners can truly feedback the problems that occur in the actual operation of DCTs, and intuitively feel the application needs and challenges. At the same time, there was relatively little research on DCTs in China, and it was unclear how practitioners view and adapt to this emerging model. The aim of this study was to investigate the knowledge, practices, and attitudes of clinical trial practitioners, including CRAs, CRCs, and PMs, toward DCTs, and to provide reference for policy makers, corporate clinical trial decision makers, and managers to promote the application and development of DCTs in China.

## 2 Methods

### 2.1 Questionnaire design

Based on the “Technical Guidance for Implementing Patient-Centered Drug Clinical Trials (Trial)” (16) released by CDE in July 2023 and the DCT element activities identified in international advanced experience (18–20), clinical research experts and senior industry practitioners were consulted, and the final decision was made through small-scale pre-research to ensure that the questionnaire content was in line with Chinese policy orientation and industry norms. The resulting in a Cronbach’s alpha coefficient value of 0.906, indicating good internal consistency.

The content of the questionnaire consisted of four parts. The first part was the basic information of the investigators (gender, age, occupation, years of employment, nature of work unit, work region). The second part was the basic knowledge of DCTs (4 questions in total). For each question, “know” got 1 point, and “do not know” got 0 point. The third part was the practice of the respondents (including 1 single choice question and 1 multiple choice question). The fourth part was the attitudes toward participating in DCTs, including the benefit factors and the obstacle factors (21 questions in total). The attitude part of the questionnaire specified response options along a 5-point Likert scale, graded from 1 (completely unimportant/very slightly) to 5 (very important/very serious) (see Supplementary material).

### 2.2 Data collection

A online survey was conducted from November to December 2023 for practitioners involved in clinical trials. Participation was both voluntary and anonymous. The questionnaire, hosted on the online platform Questionnaire Star,^1^ required approximately 4–5 min to complete. It was distributed through WeChat online communities and social media, Submission of the questionnaire was only possible after all questions were fully completed as per the online requirements. A total of 654 questionnaires were initially collected, of which 621 were deemed valid following double verification and comparison, yielding an effective response rate of 95.0%. This study received ethical approval from the Ethics Committee of North Sichuan Medical College of China.

### 2.3 Data analysis

Attitude scores were combined into categorical variables for analysis. Scores 1, 2, 3 were considered not important/not serious, and scores 4, 5 were considered important/serious. Categorical variables were expressed as n (%), and differences between CRAs, CRCs, and PMs were tested by chi-square. Binary logistic regression analysis was used to assess the association between willingness and different demographic characteristics. In this model, willingness is used as the dependent variable to analyze the influence of five main characteristics of the study population (gender, years of experience, occupation, enterprise type, and work location) on willingness in implementing DCTs. Excel and SPSS 26.0 statistical software were used for data entry, clearing and statistical analysis. Results with *p* < 0.05 were considered statistically significant.

## 3 Results

### 3.1 Demographic characteristics

This study involved 621 participants including 227 CRAs, 150 CRCs, and 244 PMs (Table 1). Among the respondents, 53.5% were male and most were between 20 and 30 years (52.3%) and 30–40 years (40.7%). The highest percentage of CRAs were those who had 1–2 years of work experience (35.7%), while most CRCs had 3–4 years (40.0%). A significantly higher proportion of PMs had more than 5 years of work experience (47.5%) compared to the other two groups. Additionally, a considerable proportion of respondents were employed by domestic pharmaceutical enterprises (28.3%) and domestic CROs (32.4%). Respondents were mainly from Beijing, Shanghai and Guangzhou (40.3%) and other provincial capitals (including Shenzhen and Suzhou) (50.4%).

**Table 1:** Demographic characteristics of respondents (*n* = 621).

Items	All (*n* = 621)		CRA (*n* = 227)		CRC (*n* = 150)		PM (*n* = 244)		*p*-value
Gender									
Male	332	53.5%	122	53.7%	60	40.0%	150	61.5%	0
Female	289	46.5%	105	46.3%	90	60.0%	94	38.5%	
Age (years)									
20–30	325	52.3%	164	72.2%	86	57.3%	75	30.7%	0
31–40	253	40.7%	55	24.2%	58	38.7%	140	57.4%	
41–50	37	6.0%	6	2.6%	4	2.7%	27	11.1%	
>50	6	1.0%	2	0.9%	2	1.3%	2	0.8%	
Years of experience									
<1	45	7.2%	28	12.3%	14	9.3%	3	1.2%	0
1–2	171	27.5%	81	35.7%	50	33.3%	40	16.4%	
3–4	210	33.8%	65	28.6%	60	40.0%	85	34.8%	
5–10	160	25.8%	47	20.7%	22	14.7%	91	37.3%	
>10	35	5.6%	6	2.6%	4	2.7%	25	10.2%	
Enterprise type									
Domestic pharmaceutical enterprises	176	28.3%	96	42.3%	16	10.7%	64	26.2%	0
Foreign pharmaceutical enterprise	121	19.5%	34	15.0%	26	17.3%	61	25.0%	
Domestic CRO	201	32.4%	81	35.7%	32	21.3%	88	36.1%	
Foreign CRO	52	8.4%	16	7.0%	7	4.7%	29	11.9%	
SMO	71	11.4%	0	0.0%	69	46.0%	2	0.8%	
Work location									
Beijing, Shanghai and Guangzhou	250	40.3%	94	41.4%	48	32.0%	108	44.3%	0.064
Other provincial capitals	313	50.4%	111	48.9%	91	60.7%	111	45.5%	
Other cities	58	9.3%	22	9.7%	11	7.3%	25	10.2%	

### 3.2 Knowledge on DCTs

**Figure 1:**
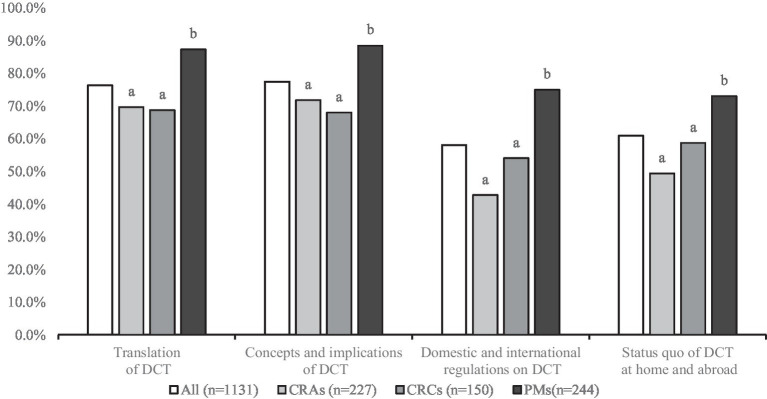
DCT knowledge among respondents (different letters indicate significant differences, *p* < 0.05).

As shown in the Figure 1, the awareness rate of “Concepts and implications of DCT” was the highest (77.5%), followed by “Translation of DCT” (76.3%) (Figure 1). However, only 58.1% of the respondents knew “Domestic and international regulations on DCT.” The awareness rate of PMs was higher than that of CRAs and CRCs, and the difference was statistically significant (*p* < 0.05).

### 3.3 Practice on DCTs

**Figure 2:**
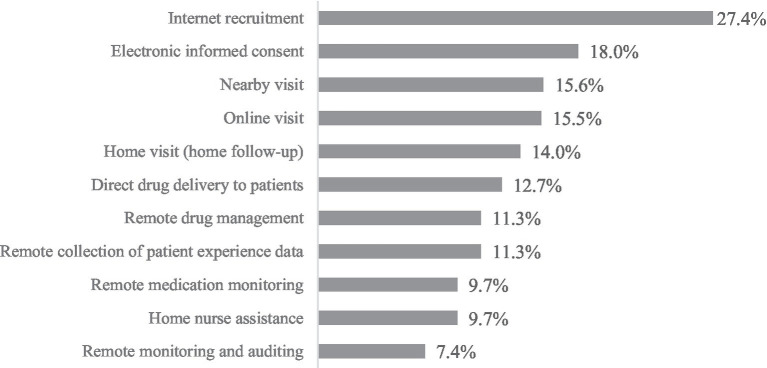
Activities of DCT among respondents.

The practical results showed that 54.8% respondents had practical experience in DCT elements, among which “internet recruitment” was the most commonly used DCT activity (27.4%), followed by “electronic informed consent” (18.0%) and “nearby visits” (15.6%). The occurrence frequency of “remote monitoring and auditing” (7.4%), “home nurse assistance” (9.7%), and “remote medication monitoring” (9.7%) were relatively low, as shown in Figure 2.

### 3.4 Attitude on DCTs

The willingness of the surveyed practitioners to participate in DCT was Relatively high (78.6%). At the same time, PMs willingness (84.8%) was higher than CRCs (76.7%) and CRAs (73.1%).

#### 3.4.1 Benefits of conducting DCTs

The evaluation of the beneficial factors for implementing DCTs was shown in Table 2. Overall, the most important item was “more clinical trial opportunities” (80.5%), followed by “improving patient experience” (80.2%) and “reducing patient burden” (80.0%), while “the emphasis on improving data quality” (71.8%) and “improving patient compliance” (73.1%) were relatively low.

**Table 2:** The benefits of conducting DCTs.

Items	All (*n* = 621)	CRA (*n* = 227)	CRC (*n* = 150)	PM (*n* = 244)	*p*-value
More clinical trial opportunities	500 (80.5)	178 (78.4)	118 (78.7)	204 (83.6)	0.293
Improving patient experience	498 (80.2)	169 (74.4)	119 (79.3)	210 (86.1)	0.006
Reducing patient burden	497 (80.0)	167 (73.6)	123 (82.0)	207 (84.8)	0.007
Improving R&D efficiency	487 (78.4)	175 (77.1)	111 (74.0)	201 (82.4)	0.121
Improving data collection efficiency	480 (77.3)	169 (74.4)	112 (74.7)	199 (81.6)	0.125
Improving safety monitoring capabilities	476 (76.7)	167 (73.6)	111 (74.0)	198 (81.1)	0.103
Increasing patient diversity	470 (75.7)	161 (70.9)	109 (72.7)	200 (82.0)	0.012
Promoting patient recruitment and retention	468 (75.4)	157 (69.2)	112 (74.7)	199 (81.6)	0.008
Reducing R&D costs	463 (74.6)	163 (71.8)	106 (70.7)	194 (79.4)	0.072
Improving patient compliance	454 (73.1)	149 (65.6)	110 (73.3)	195 (79.9)	0.002
Improving data quality	446 (71.8)	153 (67.4)	101 (67.3)	192 (78.7)	0.009

R&D indicates research and development.

For CRAs, the most recognized benefits were “more clinical trial opportunities” (78.4%), “improving (research and development, R&D) efficiency” (77.1%), and “improving patient experience” (74.4%). For CRCs and PMs, the most recognized benefits were similar to the overall situation.

#### 3.4.2 Obstacles of conducting DCTs

The evaluation of the hindering factors to participation in DCTs was shown in Table 3. Overall, respondents believed that the most serious obstacle to implementing DCTs was “the technical compatibility issues” (54.1%), followed by “data reliability and accuracy” (53.8%), and “lack of guidance from DCT related regulations” (52.5%). The barriers to “low cost-effectiveness” (45.1%) and “patient willingness and burden” (45.6%) were relatively low.

**Table 3:** Obstacles to conducting DCTs.

Items	All (*n* = 621)	CRA (*n* = 227)	CRC (*n* = 150)	PM (*n* = 244)	*p*-value
Technical barriers and compatibility issues	336 (54.1)	119 (52.4)	68 (45.3)	149 (61.9)	0.008
Reliability and accuracy of data	334 (53.8)	115 (50.7)	64 (42.7)	155 (63.5)	0
Lack of DCT related regulations	326 (52.5)	125 (55.1)	63 (42.0)	138 (56.6)	0.012
willingness and burden of researcher	325 (52.3)	111 (48.9)	67 (44.7)	147 (60.2)	0.005
Subject privacy and data security protection	324 (52.2)	120 (52.9)	65 (43.3)	139 (57.0)	0.030
Willingness and burden of the sponsor	311 (50.1)	104 (45.8)	66 (44.0)	141 (57.8)	0.008
Learning and investment costs	306 (49,3)	105 (46.3)	72 (48.0)	129 (52.9)	0.335
Willingness and burden of patient	283 (45.6)	91 (40.1)	56 (37.3)	136 (55.7)	0
Not cost-effective	280 (45.1)	97 (42.7)	55 (36.7)	128 (52.5)	0.006

#### 3.4.3 Logistic regression analysis of positive factors influencing DCTs

The results of binary Logistic regression analysis on the enthusiasm of clinical trial practitioners to carry out DCTs were shown in Table 4. The regression model was statistically significant (*p* < 0.001). Occupation and Enterprise type have significant differences in the DCT positivity, and other factors have no significant differences.

**Table 4:** Logistic regression analysis of the willingness of clinical trial practitioners.

Items	*β*	S.E	Wals	*P*	Exp (B)
Gender					
Male	1			–	1
Female	−0.165	0.218	0.574	0.449	0.848
Years of experience					
<1	1		8.268	–	1
1–2	−0.229	0.406	0.317	0.573	0.796
3–4	−0.124	0.406	0.094	0.76	0.883
5–10	0.409	0.44	0.864	0.353	1.506
>10	−0.775	0.55	1.982	0.159	0.461
Occupation					
CRA	1		16.751	–	1
CRC	1.276	0.391	10.661	0.001*	3.584
PM	0.861	0.258	11.102	0.001*	2.366
Enterprise type					
Domestic pharmaceutical enterprises	1		23.758	–	1
Foreign pharmaceutical enterprise	−0.63	0.338	3.479	0.062	0.533
Domestic CRO	−0.693	0.291	5.667	0.017*	0.5
Foreign CRO	−1.346	0.394	11.66	0.001*	0.26
SMO	−2.155	0.494	19	0*	0.116
Work location					
Beijing, Shanghai and Guangzhou	1		2.349	–	1
Other provincial capitals	−0.479	0.342	2.329	0.127	1.684
Other cities	−0.573	0.333	1.639	0.2	1.532

*p < 0.05.

## 4 Discussion

To the best of our knowledge, this is the first large-scale survey in China examining the knowledge, practices, and attitudes toward DCT. The results revealed that while the majority of respondents possessed basic knowledge of DCT and demonstrated a strong willingness to engage in them, their practical experience was limited. Additionally, some obstacles to the effective implementation of DCTs were identified.

The results of knowledge assessment showed that most respondents only had some basic understanding of DCTs. Respondents had a higher understanding of the connotation and concept of DCTs, but a lower understanding of relevant laws and regulations at home and abroad. This may be due to the fact that regulations and guidance clarifying DCT in China are still very limited, leading to a lag in practitioners’ knowledge, and on the other hand, respondents may lack practical experience with DCT, leading to relatively limited knowledge. In addition, research showed that PMs outperform CRAs and CRCS, which may be due to the fact that PMs generally have more work experience and higher job requirements.

The results of the practical investigation showed that the respondents’ participation in DCT activities was relatively low, and the application of digital health technologies was largely consistent with Daly’s global survey findings (21). This could be attributed to several factors. Primarily, the application scope of DCT was mainly confined to specific fields, regions, and projects led by leading pharmaceutical enterprises. Additionally, given the actual situation in China, implementing practices such as telemedicine, home nurse assistance, and remote data collection still faced significant challenges. Although telemedicine was considered the core of DCTs (22), telemedicine in China is currently mainly used for medical diagnosis and treatment.

The results of the practice investigation also found that Internet recruitment was relatively widely used in DCT elements, and had been widely used in China through WeChat, recruitment websites, mobile applications, network communities and other channels. A study based on ClinicalTrials.gov, a clinical trial protocol, showed that decentralized activities were primarily used for data collection (23). The comparison of these two situations reflected that during DCT’s development, different countries and regions chose diverse development priorities and paths based on their actual conditions and needs. This was crucial for promoting the diversified development of global DCT.

The attitude assessment results showed that respondents had a higher willingness to decentralize the approach to clinical trials. Regression analysis showed that occupation and enterprise type were significant factors in the willingness to implement DCT. Among the occupation, CRCs showed the highest willingness. This might be because DCT can reduce the number of patients visiting the hospital, alleviating some of the CRCs’ routine tasks, such as patient visit (24). In contrast, CRAs were relatively less proactive. Their work mainly focuses on – site monitoring and data verification. The introduction of DCT required them to learn new techniques and working methods, and will also shift some new burdens (25). Among different types of enterprises, domestic pharmaceutical companies showed the highest willingness. Currently, Chinese pharmaceutical enterprises face intense competition in the domestic market. To boost R&D efficiency and shorten the time to market, they often adopt innovative experimental methods like DCTs (26). Meanwhile, the Chinese government’s supportive policies for local pharmaceutical enterprises might also encourage them to be more willing to try new clinical trial models, aiming to secure policy support and gain a market edge (27).

In the discussion of the benefits of implementing DCTs in this study, we found that respondents were more concerned about improvements in patient rights, consistent with the findings of a fully dispersed clinical trial study in Singapore (28). DCT uses innovative technologies to cover a wider population (such as remote areas and patients with mobility difficulties), and uses telemedicine and other means to reduce the participation threshold and economic time costs, significantly improving patient convenience and experience.

Besides the focus on improving patients’ rights, improving data collection and R&D efficiency was also highly valued by clinical trial practitioners. Suman found that patients might be more proactive in remote data collection compared to traditional data collection in medical institutions (29). In the DCT model, the way patients participate in data collection has changed. They can use smart devices and mobile terminals to record and upload data, which gives patients more autonomy and convenience.

The results of this study on the implementation barriers of DCTs indicated that technical barriers and compatibility issues was the most significant obstacles to DCT implementation. Although DCTs offer numerous advantages, they also introduce new challenges, including the digital divide, data quality concerns, and the need to build new infrastructure (30). Despite these challenges, one survey found that respondents still recognize the potential of DCTs to enhance the generalizability of research findings (31).

According to the survey results, China should adopt a comprehensive approach to advancing DCTs by integrating international best practices with localized strategies. Policymakers should prioritize the development of detailed guidances to standardize remote data compliance, cross-regional collaboration, and digital ethics frameworks. Concurrently, healthcare institutions and industry stakeholders should pilot hybrid models that incrementally integrate DCT elements (e.g., telemedicine, wearable devices) into traditional trial workflows while maintaining data integrity and patient-provider relationships. To address technical gaps, a tiered training system should be established, combining online modules for foundational DCT knowledge with mentorship programs for hands-on technical skills. Additionally, creating a centralized knowledge repository to share successful case studies and regulatory updates will foster a collaborative ecosystem. By systematically addressing regulatory, operational, and educational challenges, China can accelerate the adoption of DCTs to enhance trial efficiency, patient accessibility, and global competitiveness in biomedical research.

## 5 Conclusion

In conclusion, the adoption of DCTs in China is still in its infancy. Clinical trial practitioners in enterprises generally lack extensive knowledge and practical experience with DCTs. However, their overall attitude toward these trials is positive. While respondents acknowledged the potential benefits of DCTs, they also expressed concerns about the technology, data reliability, and other related aspects.

## 6 Limitations

There are several limitations to this study. First, the study sample may not be representative of the total population due to the convenience sampling method used. Second, this study is a cross-sectional study, and therefore it is not possible to infer the evolution of relationships over time. Finally, due to the limitations of the research objectives of this paper, demographics and international differences were not examined in depth, and it is hoped that they will be further explored in future research.

## Data Availability

The raw data supporting the conclusions of this article will be made available by the authors, without undue reservation.

## Footnotes

^1^ http://www.wjx.cn/

## References

[1] Fogel DB. Factors associated with clinical trials that fail and opportunities for improving the likelihood of success: a review. Contemp Clin Trials Commun. (2018) 11:156–64. doi: 10.1016/j.conctc.2018.08.001, PMID: 30112460 PMC6092479

[2] Dockendorf MF Hansen BJ Bateman KP Moyer M Shah JK Shipley LA. Digitally enabled, patient-centric clinical trials: shifting the drug development paradigm. Clin Transl Sci. (2020) 14:445–59. doi: 10.1111/cts.12910, PMID: 33048475 PMC7993267

[3] Santa-Ana-Tellez Y Lagerwaard B De Jong AJ Gardarsdottir H Grobbee DE Hawkins K. Decentralised, patient-centric, site-less, virtual, and digital clinical trials? From confusion to consensus. Drug Discov Today. (2023) 28:103520. doi: 10.1016/j.drudis.2023.103520 36754144

[4] Dorsey ER Venuto C Venkataraman V Harris DA Kieburtz K. Novel methods and technologies for 21st-century clinical trials: a review. JAMA Neurol. (2015) 72:582–8. doi: 10.1001/jamaneurol.2014.4524, PMID: 25730665 PMC4708881

[5] European Medicines Agency. Guidance on the Management of Clinical Trials during the COVID-19 (coronavirus) pandemic. (2020). Available online at: https://www.adcreview.com/wp-content/uploads/2020/03/guidanceclinicaltrials_covid19_en.pdf (Accessed August 3, 2024).

[6] Center for Drug Evaluation. Guiding principles for the management of drug clinical trials during the COVID-19 (trial). (2020). Available online at: https://www.nmpa.gov.cn/directory/web/nmpa////xxgk/ggtg/ypggtg/ypqtggtg/20200715110101939.html (Accessed August 5, 2024).

[7] National Institutes of Health. United States: FDA and NIH issue guidance for clinical trials impacted by COVID-19. (2020). Available online at: https://clinregs.niaid.nih.gov/updates/full/6-united-states%3A-fda-and-nih-issue-guidance-for-clinical-trials-impacted-by-covid-19 (Accessed August 3, 2024).

[8] Bharucha AE Rhodes CT Boos CM Keller DA Dispenzieri A Oldenburg RP. Increased utilization of virtual visits and electronic approaches in clinical research during the COVID-19 pandemic and thereafter. Mayo Clin Proc. (2021) 96:2332–41. doi: 10.1016/j.mayocp.2021.06.022, PMID: 34481597 PMC8255101

[9] Anderson M. How the COVID-19 pandemic is changing clinical trial conduct and driving innovation in bioanalysis. Bioanalysis. (2021) 13:1195–203. doi: 10.4155/bio-2021-0107, PMID: 34275327 PMC8288280

[10] U.S. Food and Drug Administration. Decentralized clinical trials for drugs, biological products, and devices: guidance for industry, investigators, and other stakeholders. (2023). Available online at: https://www.fda.gov/media/167696/download (Accessed August 6, 2024).

[11] Danish Medicines Agency. Guidance on the implementation of decentralised elements in clinical trials with medicinal products is now available. (2021). Available online at: https://laegemiddelstyrelsen.dk/en/news/2021/guidance-on-the-implementation-of-decentralised-elements-in-clinical-trials-with-medicinal-products-is-now-available (Accessed August 3, 2024).

[12] European Medicines Agency. Recommendation paper on decentralised elements in clinical trials. (2022). Available online at: https://health.ec.europa.eu/system/files/2023-03/mp_decentralised-elements_clinical-trials_rec_en.pdf (Accessed August 9, 2024).

[13] Sato T Mizumoto S Ota M Shikano M. Implementation status and consideration for the globalisation of decentralised clinical trials: a cross-sectional analysis of clinical trial databases. BMJ Open. (2023) 13:e074334. doi: 10.1136/bmjopen-2023-074334, PMID: 37821130 PMC10582843

[14] Center for Drug Evaluation. Guidance for clinical value-oriented clinical development of antitumor drugs. (2021). Available online at: https://www.cde.org.cn/main/news/viewInfoCommon/ef7bfde96c769308ad080bb7ab2f538e (Accessed August 5, 2024).

[15] Center for Drug Evaluation. Guidance for the use of real-world evidence to support drug development. (2020). Available online at: https://www.cde.org.cn/zdyz/domesticinfopage?zdyzIdCODE=db4376287cb678882a3f6c8906069582 (Accessed August 5, 2024).

[16] Center for Drug Evaluation. Technical guidance for the implementation of patient-centered drug clinical trials (trial). (2023). Available online at: https://www.cde.org.cn/main/news/viewInfoCommon/42c008e28f7004cd19b73949142380bd (Accessed August 3, 2024).

[17] Center for Drug Evaluation. Technical guidance for the application of decentralized clinical trials in the clinical research and development of rare disease drugs. (2024). Available online at: https://www.cde.org.cn/main/news/viewInfoCommon/e5b3409ea38fbc8254bb0635d004c73d (Accessed August 15, 2024).

[18] Dorsey ER Kluger B Lipset CH. The new normal in clinical trials: decentralized studies. Ann Neurol. (2020) 88:863–6. doi: 10.1002/ana.25892, PMID: 32869367

[19] Harmon DM Noseworthy PA Yao X. The digitization and decentralization of clinical trials. Mayo Clin Proc. (2023) 98:1568–78. doi: 10.1016/j.mayocp.2022.10.001, PMID: 36669937 PMC12056663

[20] De Brouwer W Patel CJ Manrai AK Rodriguez-Chavez IR Shah NR. Empowering clinical research in a decentralized world. NPJ Digital Med. (2021) 4:102. doi: 10.1038/s41746-021-00473-w, PMID: 34211085 PMC8249659

[21] Daly B Brawley OW Gospodarowicz MK Olopade OI Fashoyin-Aje L Smart VW. Remote monitoring and data collection for decentralized clinical trials. JAMA Netw Open. (2024) 7:e246228. doi: 10.1001/jamanetworkopen.2024.6228, PMID: 38607626 PMC11015350

[22] Cummins MR Soni H Ivanova J Ong T Barrera J Wilczewski H. Narrative review of telemedicine applications in decentralized research. J Clin Trans Sci. (2024) 8:e30. doi: 10.1017/cts.2024.3, PMID: 38384915 PMC10880018

[23] de Jong AJ Grupstra RJ Santa-Ana-Tellez Y Zuidgeest MGP de Boer A Gardarsdottir H. Which decentralised trial activities are reported in clinical trial protocols of drug trials initiated in 2019-2020? A cross-sectional study in ClinicalTrials.Gov. BMJ Open. (2022) 12:e063236. doi: 10.1136/bmjopen-2022-063236, PMID: 36038171 PMC9438113

[24] Van Norman GA. Decentralized clinical trials. JACC Basic Transl Sci. (2021) 6:384–7. doi: 10.1016/j.jacbts.2021.01.011, PMID: 33997523 PMC8093545

[25] Coyle J Rogers A Copland R De Paoli G MacDonald TM Mackenzie IS. Learning from remote decentralised clinical trial experiences: a qualitative analysis of interviews with trial personnel, patient representatives and other stakeholders. Br J Clin Pharmacol. (2021) 88:1031–42. doi: 10.1111/bcp.15003, PMID: 34296777 PMC9290051

[26] DiMasi JA Smith Z Oakley-Girvan I Mackinnon A Costello M Tenaerts P. Assessing the financial value of decentralized clinical trials. Ther Innov Regul Sci. (2023) 57:209–19. doi: 10.1007/s43441-022-00454-5, PMID: 36104654 PMC9473466

[27] Zhang J Jia Z Fu S Xing H. Forecast of the development trend of decentralized clinical trials in Chinese based on GM (1,1) model. Chinese J New Drugs. (2024) 33:261–9. doi: 10.3969/j.issn.1003-3734.2024.03.008

[28] Fries LR Khaled N Viveros Santos I Suniega-Tolentino E Sesing M Toh MPS. Decentralized clinical trials are better for the participants and for the planet: the case study of a double-blind randomized controlled trial in Singapore (PROMOTE study). Front Public Health. (2025) 12:1508166. doi: 10.3389/fpubh.2024.1508166, PMID: 39872102 PMC11769950

[29] Suman A van Es J Gardarsdottir H Grobbee DE Hawkins K Heath MA. A cross-sectional survey on the early impact of COVID-19 on the uptake of decentralised trial methods in the conduct of clinical trials. Trials. (2022) 23:856. doi: 10.1186/s13063-022-06706-x, PMID: 36203202 PMC9535935

[30] Goodson N Wicks P Morgan J Hashem L Callinan S Reites J. Opportunities and counterintuitive challenges for decentralized clinical trials to broaden participant inclusion. NPJ Digital Med. (2022) 5:58. doi: 10.1038/s41746-022-00603-y, PMID: 35513479 PMC9072305

[31] de Jong AJ van Rijssel TI Zuidgeest MGP van Thiel G Askin S Fons-Martinez J. Opportunities and challenges for decentralized clinical trials: European regulators’ perspective. Clin Pharmacol Ther. (2022) 112:344–52. doi: 10.1002/cpt.2628, PMID: 35488483 PMC9540149

