# Supplementary material for: Study on knowledge, practices, and attitudes toward decentralized clinical trials among the clinical trial practitioners in China
Source: Front Med (Lausanne). 2025 Apr 17;12:1513975. doi: 10.3389/fmed.2025.1513975 (PMC12043678; doi:10.3389/fmed.2025.1513975)
Supplement: Supplementary file 1 [file Data_Sheet_1.docx]

**Questionnaire Survey on the Practices and Attitudes of Clinical Trial Practitioners towards Decentralized Clinical Trials [2023]**

Dear Sir/Madam,

Thank you very much for taking the precious time to participate in our survey. This survey aims to understand the current situation of clinical trial practitioners in China (practitioners whose occupations are related to clinical trials, including but not limited to CRAs, CRCs, clinical trial project managers, data managers, statistical analysts, clinical trial institution staff, clinical researchers, etc.) regarding their awareness, practices, and attitudes towards decentralized clinical trials, reflect the current situation and existing problems of decentralized clinical trials in China, and provide decision - making references for policymakers and industry managers in the field of decentralized clinical trials in China.

This survey will take you about 4 - 5 minutes to complete. Your honest opinions and viewpoints will determine the quality and effectiveness of the survey results. Please answer each question in the questionnaire based on the actual situation. We promise to keep your personal information confidential. This survey research is only used for academic research and industry reports and has no commercial purposes. The survey results will be announced to the whole industry through online and offline channels.

Research Center for Drug Policy and Evaluation, School of Pharmacy,

Chongqing Medical University

October 2023

**Part 1: Informed Consent**

We promise to keep your personal information confidential. This survey research is only used for academic research and industry reports and has no commercial purposes. The survey results will be announced to the whole industry through online and offline channels. Do you agree to read carefully and fill in the questionnaire truthfully? [Single - choice question]

○Yes

○No (Please skip to the end of the questionnaire and submit the answer sheet)

**Part 2: Basic Information**

1. Your gender [Single - choice question]

○Male

○Female

2. Your age [Single - choice question]

○20 - 30 years old

○31 - 40 years old

○41 - 50 years old

○Over 50 years old

3. Your occupation [Single - choice question]

○CRA

○CRC

○Project manager

○Others, please specify _________________ *

4. Your years of experience [Single - choice question]

○Less than 1 year

○1 - 2 years

○3 - 4 years

○5 - 10 years

○Over 10 years

5. The nature of your work unit [Single - choice question]

○Domestic pharmaceutical enterprise

○Foreign - invested pharmaceutical enterprise

○Domestic CRO

○Foreign - invested CRO

○Medical institution

○SMO

○Others, please specify _________________ *

6. Your working region [Fill - in - the - blank question]

_________________________________

**Part 3: knowledge**

1. Do you know the English translation of "decentralized clinical trial"? [Single - choice question]

○Yes

○No

2. Are you clear about the concept and connotation of decentralized clinical research? [Single - choice question]

○Clear

○Unclear

3. Are you familiar with the domestic and foreign regulations on decentralized clinical trials? [Single - choice question]

○Familiar

○Unfamiliar

4. Do you understand the current situation of decentralized clinical trials at home and abroad? [Single - choice question]

○Understand

○Don't understand

**Part 4: Practices**

1. Have you participated in a clinical trial that uses decentralized technologies/means? [Single - choice question]

○Yes

○No (Please skip to question 13)

2. Which of the following decentralized technologies/means were used in the decentralized clinical trials you participated in? [Multiple - choice question]

□Recruitment through the Internet platform

□Electronic informed consent

□Nearby visits (substitute visits, transfer of subjects)

□Home visits (home follow - up)

□Online visits

□Direct drug delivery to patients

□Remote drug management

□Nurses visiting to assist

□Medication reminders/ compliance monitoring

□Remote collection of patient - reported outcomes (smart devices, mobile terminals)

□Remote monitoring and auditing

**Part 5: Attitudes**

1. What is your overall attitude towards participating in clinical trials using decentralized means? [Single - choice question]

○Very positive

○Positive

○Generally positive

○Not positive

○Completely negative

2. Your attitude towards the benefits of applying decentralized clinical trials: [Matrix single - choice question]

**very important, important, generally important, not important, completely unimportant**

Improving R&D efficiency

Improving data quality

Reducing R&D costs

Improving patient compliance

Promoting patient recruitment and retention

More clinical trial opportunities

Improving patient experience

Reducing patient burden

Improving safety monitoring capabilities

Improving data collection efficiency

Increasing patient diversity

3. Your attitude towards the obstacles of applying decentralized clinical trials: [Matrix single - choice question]

**very serious, serious, generally serious, not serious, very slightly**

Reliability and accuracy of data

Willingness and burden of the sponsor

Willingness and burden of patient

willingness and burden of researcher

Subject privacy and data security protection

Learning and investment costs

Technical barriers and compatibility issues

Not cost-effective

Lack of DCT - related regulations
